# Paraneoplastic stiff person syndrome associated with colon cancer misdiagnosed as idiopathic Parkinson’s disease worsened after capecitabine therapy

**DOI:** 10.1186/1477-7819-11-224

**Published:** 2013-09-12

**Authors:** Sasa Badzek, Vladimir Miletic, Juraj Prejac, Irma Gorsic, Hilda Golem, Ervina Bilic, Domina Kekez, Niksa Librenjak, Stjepko Plestina

**Affiliations:** 1 Department of Oncology, Division of Gastrointestinal Malignancies, University Hospital Center Zagreb, Kispaticeva 12, 10000 Zagreb, Croatia; 2 Department of Neurology, Movement Disorders Centre, University Hospital Center Zagreb, Kispaticeva 12, 10000 Zagreb, Croatia

## Abstract

**Objectives:**

To refresh clinical diagnostic dilemmas in patients presenting with symptoms resembling to those of parkinsonism, to report rare association of colon cancer and paraneoplastic stiff person syndrome (SPS), and to draw attention on the possible correlation of capecitabine therapy with worsening of paraneoplastic SPS.

**Methods:**

Case report of the patient with paraneoplastic SPS due to colon cancer that was misdiagnosed as idiopathic Parkinson’s disease (iPD), whose symptoms worsened after beginning adjuvant capecitabine chemotherapy.

**Results:**

We describe a 55-year-old woman with subacute onset of symmetrical stiffness and rigidity of the truncal and proximal lower limb muscles that caused lower body bradykinesia, gait difficulties, and postural instability. Diagnose of iPD was made and levodopa treatment was initiated but failed to provide beneficial effect. Six months later, colon cancer was discovered and the patient underwent surgical procedure and chemotherapy with capecitabine thereafter. Aggravation of stiffness, rigidity, and low back pain was observed after the first chemotherapy cycle and capecitabine was discontinued. Furthermore, levodopa was slowly discontinued and low dose of diazepam was administered which resulted in partial resolution of the patient’s symptoms.

**Conclusion:**

Paraneoplastic SPS is rare disorder with clinical features resembling those of parkinsonian syndrome and making the correct diagnosis remains a challenge. The diagnosis of parkinsonian syndrome should be re-examined if subsequent examinations discover an associated malignant process. Although it remains unclear whether the patients with history of SPS are at the greater risk for symptoms deterioration after administration of capecitabine, clinicians should be aware of capecitabine side effects because recognition and appropriate management can prevent serious adverse outcomes.

## Background

Stiff person syndrome (SPS) is a rare immune-mediated disorder, which was first described by Moersch and Woltman in 1956 [1]. Classic SPS is characterized by mostly symmetric muscle stiffness and rigidity in axial and proximal limb muscles, intermittent spasms, and pronounced sensitivity to various external or emotional stimuli [2,3]. Affection of lumbar paraspinal muscles causes typical lumbar hyperlordosis that persists in supine position but diminishes during sleep. Impaired truncal and leg mobility cause walking difficulties with a slow, stiff-legged gait. Although the exact pathogenesis of SPS is still unclear, autoantibodies against glutamic acid decarboxylase (GAD), the enzyme responsible for the synthesis of γ-aminobutyric acid (GABA), are found in approximately 60% to 80% of SPS patients, thus suggesting important role of immune system in the etiology of SPS [4,5]. In addition, autoimmune disorders such as diabetes, thyroiditis, and pernicious anemia occur more frequently in patients with SPS. It is assumed that decreased GABA-ergic inhibition at brainstem and spinal cord level is responsible for enhanced motor excitability [6]. SPS can occur also as a paraneoplastic condition. Five percent of all SPS patients are associated malignancies [7]. Association of colon cancer and SPS has rarely been reported. Here we report a case of paraneoplastic SPS due to colon cancer that was misdiagnosed as idiopathic Parkinson’s disease (iPD) with a worsening of symptoms after inducing capecitabine in therapy.

## Case presentation

A 55-year-old woman was examined by a neurologist in March 2011 because of subacute onset truncal and lower limb stiffness, bradykinesia, and gait difficulties. Her past medical history included arterial hypertension, hysterectomy with bilateral adnexectomy due to cervical cancer in 1999, consequent osteoporosis, and thyroidectomy due to Hürthle cell adenoma in 2007. In September 2007 she was hospitalized because of lacunar ischemic stroke in pons with mild gait ataxia as a sequel. There was no prior history of parkinsonism, carbon monoxide poisoning, CNS infection, neuroleptic and toxin exposure. Routine laboratory tests, CBC, ESR, CRP, liver function tests, electrolyte panel, coagulation profile, triiodothyronine, thyroxine, and thyroid-stimulating hormone were within normal limits. 123I-ioflupane (DaTSCAN) imaging was also performed, and showed no signs of nigrostriatal dopaminergic abnormalities. However, the diagnosis of iPD was made. Rasagiline and levodopa were initiated but failed to provide beneficial effect. On the contrary, her symptoms worsened gradually. Six months later, sigmoid colon cancer was discovered as an underlying cause of newly discovered iron deficiency anemia. Left hemicolectomy was performed in September 2012. Pathological review revealed colon adenocarcinoma, pT3N0M0, with intermediate differentiation and microperforations as adverse features, and adjuvant chemotherapy with capecitabine was started in September 2012. Aggravation of stiffness, rigidity, and general mobility was observed after the first cycle, and capecitabine was discontinued. The patient was referred to a Movement Disorders Centre for a second opinion. Examination revealed stiffness and rigidity of the truncal and proximal lower limb muscles and pronounced lumbar hyperlordosis. Her gait was stiff-legged and wide-based, but deductible only with the assistance of another person. No signs of upper limb bradykinesia were found. At the time, the patient was practically wheelchair-bound. High suspicion of paraneoplastic SPS was made and levodopa was slowly discontinued, while a dose of 10 mg diazepam BID was initiated. In addition, analysis of serum anti-GAD antibody and onconeural antibodies (Hu, Ri, Yo, Amphiphysin) was made, but was negative, and levels of vitamin B12 and folic acid were within the normal range. Therapy modification resulted in significant improvement of patient’s symptoms and daily functioning. An additional movie file shows this in more detail (see Additional file 1). As recommended by her oncologist, adjuvant chemotherapy was continued for 6 months total with simplified biweekly infusional 5-fluorouracil plus leucovorin (sLV5FU2) according to recent NCCN guidelines, without further neurological deterioration [8].

## Conclusions

According to the clinical picture and course of the disease, our patient fulfilled criteria for paraneoplastic SPS, although no onconeuronal antibodies were detected [9]. We find our case interesting for several reasons. First, clinical features of paraneoplastic SPS can resemble those of parkinsonian syndrome and making the correct diagnosis can be challenging. It is hard to explain how SPS has not been recognized earlier, but we speculate that rarity of the condition could be attributed as a most likely case scenario. Therefore, we would like to emphasize that the diagnosis of iPD should be re-examined in patients with subacute debut, rapid progression of symptoms, lack of efficiency of dopaminergic therapy, and atypical symptoms. Bearing in mind that published data exist supporting the association of seronegative SPS and cancer in about 25% of cases, although with no causal relationship proven, in such patients additional diagnostic procedures are required and possible underlying malignancies should be excluded [10]. In addition, the diagnosis of parkinsonian syndrome should be re-examined if subsequent examinations discover an associated malignant process. Second, paraneoplastic SPS usually occur in the presence of breast cancer, small cell lung carcinoma, thymoma, and Hodgkin’s lymphoma, and so far, we found only one report similar to ours [11]. This association should be kept in mind, because early-stage tumor detection enables appropriate treatment. And third, clinical observation of aggravation of stiffness, rigidity, and low back pain with capecitabine therapy should not be ignored. Low back pain is known to be a possible side effect of capecitabine, but whether the patients with history of SPS are at greater risk for symptom deterioration remains unclear, and more reports are needed for providing any conclusion. A possible explanation could be the interaction of capecitabine and its metabolites with inhibitory GABA-ergic pathway.

## Consent

Written informed consent was obtained from the patient for publication of this case report and any accompanying images. A copy of the written consent is available for review by the Editor-in-Chief of this journal.

## Competing interests

The authors declare that they have no competing interests.

## Authors’ contributions

SB and VM have drafted the manuscript, carried out diagnostic studies, and guided the patients’ therapy. EB has performed neurological diagnostic studies. JP, IG, HG, DK, NL, and SP have been involved in drafting the manuscript. All authors read and approved the final manuscript.

## Supplementary Material

Additional file 1 Paraneoplastic stiff person syndrome. Click here for file
